# Dementia and cognitive impairment in adults as sequels of HSV-1-related encephalitis: a review

**DOI:** 10.1590/1980-57642021dn15-020002

**Published:** 2021

**Authors:** Emille Magalhães Neves Campos, Laís Damasceno Rodrigues, Leandro Freitas Oliveira, Júlio César Claudino dos Santos

**Affiliations:** 1 Faculty of Medicine, Federal University of Roraima, Boa Vista, RR, Brazil.; 2 Neuroscience Laboratory, Department of Neurology and Neurosurgery, Federal University of São Paulo, São Paulo, SP, Brazil.; 3 Faculty of Medicine, Christus University Center, Fortaleza, CE, Brazil.

**Keywords:** herpesvirus 1, human, central nervous system viral diseases, encephalitis, herpes simplex, cognitive dysfunction, dementia, herpesvírus humano 1, viroses do sistema nervoso central, encefalite por herpes simples, disfunção cognitiva, demência

## Abstract

Considering the variety of mechanisms of Herpes simplex virus (HSV-1) contamination and its broad invasive potential of the nervous system, a life-long latent infection is established. Infected adult individuals may be susceptible to viral reactivation when under the influence of multiple stressors, especially regarding immunocompromised patients. This guides a series of neuroinflammatory events on the cerebral cortex, culminating, rarely, in encephalitis and cytotoxic / vasogenic brain edema. A sum of studies of such processes provides an explanation, even though not yet completely clarified, on how the clinical evolution to cognitive impairment and dementia might be enabled. In addition, it is of extreme importance to recognize the current dementia and cognitive deficit worldwide panorama. The aim of this literature review is to elucidate the available data upon the pathophysiology of HSV-1 infection as well as to describe the clinical panorama of the referred afflictions.

## RESUMO.

Considerando a variedade de mecanismos de contaminação pelo vírus Herpes simplex (HSV-1) e seu amplo potencial invasivo do sistema nervoso, uma infecção latente por toda a vida é estabelecida. Indivíduos adultos infectados podem ser suscetíveis à reativação viral quando estão sob a influência de múltiplos estressores, principalmente em pacientes imunocomprometidos. Esse fator orienta uma série de eventos neuroinflamatórios no córtex cerebral, culminando, raramente, em encefalite e edema cerebral citotóxico/vasogênico. Um somatório de estudos desses processos fornece uma explanação, embora ainda não totalmente esclarecida, de como a evolução clínica para déficit cognitivo e demência pode ser possibilitada. Além disso, é de extrema importância reconhecer o panorama mundial atual da demência e do déficit cognitivo. O objetivo da presente revisão de literatura é elucidar os dados disponíveis sobre a fisiopatologia da infecção pelo HSV-1, assim como descrever o panorama clínico das referidas afecções.

## INTRODUCTION

The herpes simplex virus type 1 (HSV-1) is a double-stranded DNA virus and belongs to the Alphaherpesvirinae subfamily,^1^ whose genome encodes more than 80 different open reading frames. Characterized by a short intracellular replication cycle, with rapid destruction of the host cell, HSV-1 can alternate between an infectious lytic phase, producer of infectious virions, and a latent state, which enables a long-term persistence of infection, dissemination and escape from the immune system surveillance.^2^

Its broad neurotropic potential dictates a wide spectrum of clinical disorders, ranging from simpler mucocutaneous manifestations, such as oral and facial injuries, to serious infections of the central nervous system (CNS). Among these, HSVs account for 50 to 75% of viral encephalitis, which consist of brain parenchyma inflammation associated with clinical evidence of neurologic dysfunctions, such as reduced consciousness, behavioral alterations, and impaired cognitive function.^3^ The herpetic encephalitis (HSE) is responsible for a high mortality rate between patients who do not receive antiherpetic treatment and for over 70% of neurological sequelae in surviving patients.^3^

Given the HSV-1 wide invasive potential of the CNS, it is able not only to agglomerate in the dorsal root nerve ganglia (DRG), but also to migrate to the cerebral cortex itself, including the orbitofrontal, insular, and mesial temporal lobe regions, specially affecting the hippocampus,^4^ whose vulnerability to Alzheimer disease is substantial, as demonstrated in pioneer and recent studies.^5^ ^,^ ^6^ ^,^ ^7^ Furthermore, evidence carried out with animal models prove that the area analogous to the human limbic cortex is commonly affected by encephalitis. A contralateral temporal lobe spread may as well occur via the anterior commissure.^8^

**Figure 1.**
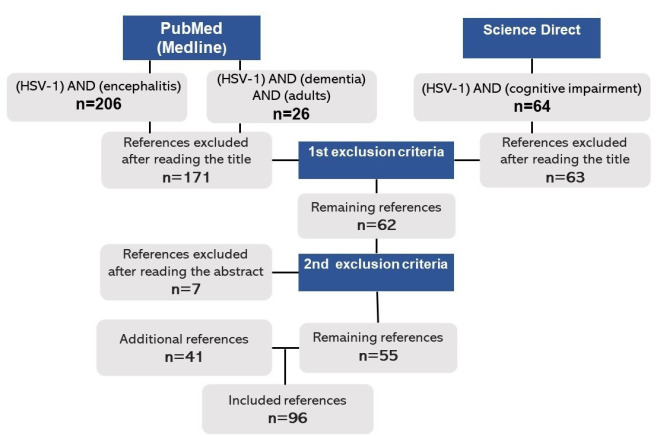
Articles addressing to HSV-1 infection and Alzheimer disease/neurodegeneration.

AD: Alzheimer disease; CNS: central nervous system; APOE4: apolipoprotein E4; APP: amyloid precursor protein; aMCI: amnestic mild cognitive impairment; A(: amyloid-beta plaques.

Most cases of encephalitis in adults are related to viral infection by HSV-1.^9^ This scenario enables the clinical evolution to neuroinflammatory and glial damage processes,^10^ mostly threatening immunocompromised or immunosuppressed patients, whose possible unfavorable prognosis are cognitive impairment and dementia.^11^ ^,^ ^12^ Such entities, nevertheless, are also likely to occur in infected individuals without previous encephalitis.^13^ ^,^ ^14^ ^,^ ^15^ These disorders are widely correlated in several studies on the pathophysiology of Alzheimer disease in aged individuals with comorbidities, but are scarce among healthy adult individuals, in which the pathogenesis is still uncertain. A summary of the main works regarding HSV-1 infection and Alzheimer disease and neurodegeneration can be found in Figure 1. The aim of this literature review was to elucidate the pathophysiology of the neuroinflammation due to HSV-1 encephalitis and its clinical evolution to cognitive impairment and dementia in adults, as well as to describe the respective clinical panorama.

## METHODS

A narrative literature review was conducted in the Medline and Science Direct Databases, from 2010 to 2020, on dementia, cognitive impairment, and the HSV-1 encephalitis/infection. In the Medline Database, 206 articles were found by the Mesh descriptor “(HSV-1) AND (encephalitis)”, of which 48 were selected, after the first set of criteria - exclusion of titles not addressing to the topic ‘HSV-related encephalitis’, articles not included in the search period 2010-2020, as well as non-English articles. Following the same exclusion criteria, 13 of 26 articles were selected by the descriptor “(HSV-1) AND (dementia) AND (adults)” from the Science Direct Database. When searching the descriptor “(HSV-1) AND (cognitive impairment)” in the same database, a total of 64 articles from the year 2020 were summarized, of which 1 was selected. The second set of criteria - exclusion of the abstracts not addressed to HSV as the cause for encephalitis in adults - was applied, by which 7 articles were excluded. However, in such criteria, experiments on animal models were included due to the analogous relation between the cortical areas affected and those in the human brain. Moreover, although not being the target population, some studies with older individuals (age>60) were also included when addressing to the HSV reactivation frequency follow-up and its potential risk for Alzheimer disease. Other articles that did not contemplate these conditions were excluded. Furthermore, 41 articles from the Medline Database were manually screened and added according to their relevance in the qualitative evidence synthesis. Of the total, 96 original articles in English (including book chapters, guidelines, and case reports) remained.

## MECHANISMS OF THE HSV-1 INFECTION

Considering the mechanisms by which HSV-1 reaches the CNS for the development of HSE, some pathways are elucidated, either by primary or secondary infection.^16^ Among these routes of contamination, there is the olfactory/trigeminal tract, the hematogenous via, and the peripheral ganglia reactivation.

The first mechanism includes the oropharyngeal via toward the olfactory epithelium neurons and posteriorly retrograde axonal transport to the olfactory bulb in the brain. This type of infection usually occurs first in the axonal termini of peripheral neurons of the trigeminal ganglion (TG), which innervate the orofacial or corneal layer.^17^ Given the fact that trigeminal neurons are pseudounipolar, the ascendent pathway of one branch projects into the thalamus and sensory cortex, whereas the other branch reaches the trigeminal nucleus in the brainstem. This factor enables new viral particles to migrate to the CNS through anterograde route. Hence, trigeminal neurons have been considered as the gateway to HSV-1 in the CNS.^18^

Eventually, the hematogenous vertical transmission route is possible after previous infection of the mother and subsequent access of HSV-1 to the placenta through the bloodstream. In addition, the contamination of the newborn includes the peripartum and postnatal periods,^19^ as reported in the study carried out by Avila and coworkers in southern Brazil, in which 480 placenta and umbilical cord biopsies of postpartum women were analyzed. The prevalence of HSV-1 infection in placental DNA was 37.5% compared to 27.5% in the umbilical cord.^20^ After entering the fetal organism, the virus migrates to the brain, establishing itself in the hippocampus. The infection also extends to neural stem cells in the subventricular zone of the lateral ventricle and to the hippocampal subgranular zone, affecting cell maturation, differentiation, and proliferation.^21^

The third mechanism of viral dissemination is represented by peripheral ganglia reactivation with subsequent axonal migration to the CNS after an episode of recurrent orofacial infection by HSV-1.^22^ However, latent *in situ* reactivation of the virus in the CNS tissue is eventually found, when under the influence of stressors, such as fever, sunlight, immunosuppression, emotional stress, exposure to ultraviolet rays, hormonal changes, dental surgery, and head trauma.^23^ ^,^ ^24^ ^,^ ^25^ ^,^ ^26^

Latent HSV-1 infection is guided by many factors of the human nervous system, thereby the same viral etiology is responsible for primary infections located in the same site of cutaneous distribution in immunocompetent individuals.^27^ Viral reactivation is likely to occur at any time in the patient's life, unlike primary infection, which usually occurs between two to three decades of life.^27^ In a prospective study on risk factors for HSV-1 infection/reactivation of a British populational base, conducted by Forbes and collaborators, such incidence was correlated with the increasing age and lower educational level and socioeconomic status of the population. The female seropositive portion (64.2%), between the age group of 50-59, had higher HSV-1 infection and the highest reactivation rates, whose values ​​were 59.2 and 20.4%, respectively.^28^ This result is in accordance with the data from previous studies.^29^ ^,^ ^30^

Regardless of whether individual show symptoms after infection with HSV, the life-long infection with the virus implicates metabolic alterations in neuronal cells, increasing pathological mechanisms in the body and brain of some infected hosts.^31^ ^,^ ^32^ The physiological effects possibly culminate, among those with a worse prognosis, in herpetic encephalitis and consequent cognitive dysfunction.^12^ ^,^ ^17^ ^,^ ^27^ ^,^ ^33^

## LYTIC AND LATENT HSV-1 INFECTION: GENE MODULATION

The fusion of HSV-1 with the neuronal membrane of sensory neurons at the axonal termini enables viral spread from its primary site of infection. Its nucleocapsid then is retrogradely transported to a ganglion (usually the trigeminal ganglion) and reaches the nucleus in the cell body, where it releases its DNA in an episomal form. During lytic infection, several genes are expressed in a cascade pattern, in which the viral transcriptional protein (VP16) is responsible for the immediate-early (IE) gene expression. IE gene products are expressed after 2‒4 hours of the primary infection, *i.e.*, infected cell protein 0 (ICP0), ICP4, ICP22, ICP27, and ICP47, that consequently activate the expression of early (E) and late (L) genes and stimulate viral DNA replication. Therefore, during lytic infection, the VP16 viral protein acts not only in the general chromatin reduction, but also in the alteration of euchromatin from the histones associated with HSV lytic genes.^34^ ICP-0 and US3 viral gene products also execute important functions by inhibiting the viral genome silencing and conducting viral expression in the host.^35^

After entering sensory neurons, the congregate virus expresses latency-associated collinear transcripts (LAT), observed in the trigeminal ganglia of infected mice on the first 24‒72 hours, as well as several microRNAs (miRNA).^34^ LAT is the only abundant viral transcript that is expressed in the infected neurons and plays an important role in the silencing of lytic gene expression.^34^ These components deregulate the expression of the host’s miRNA, exploiting it to advance its replication or suppressing its genes to facilitate the state of latency and reactivation.^36^ ^,^ ^37^ ^,^ ^38^ Moreover, the existence of certain genetic compounds in some individuals (such as the SNORA31 gene) tends to facilitate the susceptibility to HSV-1’s neurovirulence.^39^

This control mechanism is also demonstrated by the study of Bhela and collaborators, when determining susceptibility to viral invasion based on the presence of miR-155 in mice models. Such miRNA confers the risk for developing an inflammatory disease by both promoting the expansion of pro-inflammatory Th1 and Th17 cells as well as amplifying effects on inflammatory gene expression in host macrophages and T cells. Thereby, when in contact with HSV-1, there is a worsening of the viral pathogenicity, represented by ocular disease, HSE, and zosteriform lesions in the host. Additionally, rapid reactivation effectiveness was observed in ganglionic latent infection with miR-155.^40^ This gene modulation has also been proven in previous studies with rodents.^41^

Latency is also maintained by several CNS immunological factors that suppress viral replication, as represented by IFN-β and its genetic modulator (y34.5)^42^ as well as the APOBEC1 gene^43^ and CD8+T cells.^44^ ^,^ ^45^ ^,^ ^46^ In spite of their apparently benign and harmless character, latent infections not only represent a potential reservoir for viral reactivation, but also that the latent virus itself can contribute to events that modify the pathophysiology of the adjacent disease.^12^ The production of reactivating virions can occur when the host’s immune system is compromised,^47^ ^,^ ^48^ ^,^ ^49^ ^,^ ^50^ with outcomes varying between local inflammatory and systemic diseases.^51^ ^,^ ^52^ ^,^ ^53^ Transplanted patients who are undergoing immunosuppressant therapy generally present a drastic increase in viral activity^54^ ^,^ ^55^ and the inflammatory stress of aging also alters immune responses, allowing the reactivation of herpesviruses (HVs).^56^

## PATHOPHYSIOLOGY OF HSV-1 ENCEPHALITIS AND NEUROINFLAMMATION INSIGHT

It is important to ratify that HSV encephalitis is a rare disease, with an incidence of 2‒4 cases per 1,000,000 individuals per year,^57^ ^,^ ^58^ ^,^ ^59^ while the trigeminal ganglion reactivation and clinical herpes labialis are common entities. Viral reactivation in the peripheral ganglia corresponds to 70% of HSE cases, whereas only 30% of cases correlate to primary HSV infection, whose frequency in children and adolescents is considerable. In both forms of infection, the clinical manifestations and evolution of HSE are the same.^60^

The presence of HSV-1 in the brain results in the continuous activation of the microglia, which contributes to a huge cytokines/chemokines secretion and to a substantial neuroinflammatory activity. Specific pattern-recognition receptors (PRR) on microglia and astrocytes, such as Toll-like receptors (TLR), recognize pathogen-associated molecular patterns (PAMP), such as proteins or nucleic acids from the virus. These PAMP cause an intense type I IFN and antiviral gene expression, which induce the production of numerous pro-inflammatory cytokines, exemplified by the tumor necrosis factor (TNF), interleukin (IL)-1, IFN-alpha, and IL-6. Chemokines produced by microglia (*e.g*., CCL5, CXCL10, MCP-1, NO) and antimicrobial proteins (*e.g*., iNOS) are also involved in the modulation of PRR signaling, whose function is to guide apoptosis, cell recruitment, and other inflammatory procedures in the tissue.^61^ The respective TLR are coupled to signaling adaptor systems, resulting in the activation of downstream kinases, consequently modulating intracellular signaling through nuclear factor κB (NF-κB) or interferon regulatory factors (IRF).^17^ The currently known TLR on microglia that identify HSV-1 components include TLR2, TLR3, and TLR9.^62^

In view of this scenario, the double-edged role of NO during an HSV-1 infection in primary neuronal cultures and in mixed glial cultures has been proven by the study of Cymerys and coworkers, according to which HSV-1 induces a high expression of IFN-alpha, TNF-alpha, CXCL9, and CXCL10 in neuronal cultures. When NO is synthesized, it downregulates the activity of certain cytokines (like IFN-alpha and IFN-beta, from Th1 response). Such cytokines act in viral replication control and *in vitro*/*in vivo* spread during primary infection and reactivation, as well as upregulating iNOS in uninfected glial cells.^63^ They also execute important roles in recruiting dendritic cells (DC), natural killer cells (NK), B, and T cells.^64^ In addition, a potent production of type I interferon (IFN) via the cGAS-STING pathway has proven to antagonize HSV-1-driven encephalitis.^65^ ^,^ ^66^

Hence, disruption of the blood-brain barrier (BBB) succeeds such neuroinflammation enhancement during HSE, leading to unfavorable outcomes like vascular brain edema, hemorrhage, and leukocyte infiltration.^67^ This mechanism is reached due to the high production and binding of cytokines (such as IL-1β and TNF-α) to ICAM-1 glycoproteins, abundantly expressed on the surface of endothelial cells of the brain vasculature when stimulated by pathogens.^68^ Mitochondrial dysfunction also implicates astrocyte apoptosis through intrinsic and extrinsic pathways,^69^ including alterations in the aquaporin 4 (AQP4) membrane transport protein, intimately involved in water translocation across the BBB, astrogliosis, cell apoptosis, and neuroinflammation. This process leads to an intra/extracellular fluid accumulation, culminating in cytotoxic and vasogenic brain edema.^69^

Piacentini and companions evidenced that HSV-1 also leads to neuronal hyperexcitability as well as altered synaptic activity, which cause dysregulation of intracellular Ca^2+^ homeostasis and β- and γ-secretases pathways, consequently increasing amyloid precursor protein (APP) processment, amyloid β (Aβ) extracellular plaques, and hyperphosphorylated tau production, found post-mortem in the brains of infected patients as intracellular neurofibrillary tangles.^70^ Markers of primary or reactivated HSV-1 infection, such as anti-HSV-1 IgM antibodies, correlate with the increased risk of AD in aged individuals, a statement obtained from a large population-based cohort study.^71^ Other studies found an association between IgG anti-NMDAR serostatus and impaired recovery of cognitive performance in individuals with HSE, present in approximately 25% of patients.^72^ ^,^ ^73^

Moreover, mitochondrial dysfunction is observed in Alzheimer disease (AD) brains, when associated with a decline in mitochondrial membrane potential in addition to reactive oxygen species (ROS) increasement.^74^ ^,^ ^75^ These processes are known to influence synaptic communication^76^ ^,^ ^77^ and rearrangements of mitochondrial DNA.^78^ Therefore, dysregulation of transcription factors, like NF-kB and AP-1, culminates in neuronal apoptosis. Proinflammatory cytokines act on cholinergic neurons and stimulate astrocytes, amplifying proinflammatory signals to induce neurotoxicity. The ATP releasement due to neuronal death subsequently stimulates microglia, an event that maintains such cyclical neuroinflammation.^79^

## DEMENTIA AND COGNITIVE DEFICIT PANORAMA: ASSOCIATION WITH HSV-1

The dementia syndrome is considered as a brain disease that compromises patients’ quality of life, family relationships, and productivity of their relatives and caregivers, representing a significant financial and psychosocial burden.^80^ Despite the existence of numerous researches upon dementia and the HSV-1 brain infection individually, the underlying pathophysiological mechanisms of association between both remain uncertain.^15^ As part of the main neuroinflammation supporters and as first line of defense against CNS infections and damage, the activated state of microglia has been linked to neurotoxicity, neurodegeneration, and chronic neuroinflammation in several disorders including Alzheimer disease, Parkinson disease, amyotrophic lateral sclerosis, and multiple sclerosis.^21^

About this perspective, several studies on the potential risk of HSV-1 infection for neurodegenerative diseases have been conducted.^6^ ^,^ ^7^ ^,^ ^21^ ^,^ ^81^ ^,^ ^82^ ^,^ ^83^ ^,^ ^84^ ^,^ ^85^ ^,^ ^86^ Focusing especially in AD susceptibility, an increased risk was confirmed for the development of the disease in the presence of the apolipoprotein E epsilon 4 (APOE4) allele, for this allele frequency is much higher between individuals infected by HSV-1 than the non-infected AD population.^87^ Impaired hippocampal neurogenesis was also observed due to A( protein production in infected adults, demonstrated by alterations in stem cell proliferation and neuronal differentiation.^88^

HSV-1 encephalitis and Alzheimer disease tend to compromise some common cortical areas that play a key role in behavior, memory, and cognition, such as the orbitofrontal cortex, insular cortex, temporal mesial lobe (especially the hippocampus), and the cingulate gyrus. Damage in those areas are strongly related to the development of neurological and psychiatric sequelae, including seizures, delusions, hallucinations, impairments in working and verbal memory, as well as in executive functions.^89^ ^,^ ^90^

Therefore, lesions in the orbitofrontal cortex, for example, are deeply related to emotional/social behavior changes, such as reversal learning, linked to behavioral disinhibition or even an ‘antisocial behavior’ and impulsiveness.^91^ The insular cortex enrollment in cognition is related to encoding the interoceptive signals that reflect autonomic activity, especially regarding fear (when interacting with the amygdala) and disgust, even though the last one is still suggestive.^92^ Plenty functional neuroimaging studies have found specific and generic emotional/behavioral effects related to the insula, which include the anterior cingulate cortex participation in tasks involving perception, intentional action, and consciousness, such as high-level cognitive control, emotional subjective awareness, and empathic and attentional processes.^92^

Regarding magnetic resonance imaging (MRI), interesting results were found in the study by Harris and collaborators, who analyzed the neuropsychological impact of HSV in short-, medium-, and long-terms (4 months; 9‒12 months, and >1 year post-discharge from the hospital, respectively). A significant impairment of both anterograde and retrograde memory as well as diminished verbal response were observed in HSV-post-infected patients. Additionally, executive functions, IQ, and naming abilities were also reduced, differing from the group with other causes of encephalitis. This last group had a moderate memory loss with a certain preservation of the executive functions. Such remarkable memory impairment was associated with hippocampal/medial temporal gyrus damage, illustrated on MRI with a substantial volume loss, including the typical hyperintensity presented on the T2-weighted sequence. A proportional correlation between the extent of temporal mesial lobe damage with the severity of anterograde/retrograde amnesia was identified. Moreover, in the short-term, higher rates of depression were noted between the HSV-encephalitis group when compared to the group with other causes of encephalitis. Both depression and anxiety, though, persisted with raised levels in all encephalitis groups in the long-term.^93^

Upon the HSV therapeutic approach, Tzeng and companions conducted a cohort study on HSV-1 and the development of dementia with 8,362 patients ≥ 50 years of age, post-infection, for a 10-year follow-up, from Taiwan’s Longitudinal Health Insurance Database. An exponential chart was formed, which estimates the cumulative risk of dementia in HSV patients over the years. Individuals were stratified into 3 groups, respectively: non-treated HSV infection; treated HSV infection; and non-infected group. Evidently, the sharpest curve was noticed among the individuals of the first group, even though those infected had a 2.56-fold greater risk of developing any type of dementia, including Alzheimer disease, vascular dementia or other dementias.^15^

Through a historical prospective study, Fruchter and collaborators analyzed the impact of HSV on the cognitive capacity of 612 healthy individuals, from a sample of soldiers of the Israeli defense force, whose serum status percentages were 62.2% HSV-positive and 38.8% HSV-negative. Cognitive function and linguistic ability of both groups were tested, and HSV seropositive soldiers expressed significantly lower IQ scores when compared to the seronegative ones, thus suggesting the link between HSV-infection and cognitive functional reduction in such individuals.^94^ Similar outcomes were also observed in previous researches with adults with schizophrenia.^89^ ^,^ ^95^ ^,^ ^96^

Corroborating the cognitive impacts caused by HSV-1 in healthy persons, Tarter and coworkers evaluated several patients from different age groups that were infected by the virus, including by Cytomegalovirus (CMV), from the Herpesviridae family. Among children (6 to 16 years old), HSV-1 seropositivity was associated with reduced reading and spatial reasoning test scores. Both HSV-1 and CMV seropositivity in middle-aged adults (20 to 59 years old) were associated with impaired cognition speed and, among the aged, with memory impairment. The data indicated that HSV-1 can impair cognition throughout life in all age groups, whose risk of deficit gradually increases the earlier the exposure to the virus takes place in lifetime.^14^

A sum of longitudinal, cross-sectional, transversal, cohort, and experimental studies has been developed upon HSV-1 and its potential risk for dementia and cognitive impairment in infected individuals. Nevertheless, the pathophysiology of the related encephalitis is still not completely understood, as many researches remain mostly suggestive regarding a causal link. A sense of urgency to conduct further studies is required, therefore, to understand the clinical evolution to neurodegeneration and lack of cognition, specifically involving adults, since the majority of available evidence tends to frame the elder population. Through understanding this process and its worldwide panorama, clinicians are able to improve their diagnosis and therapy with the aim of preventing the progression to such devastating outcomes, whenever possible.
